# Electrical and Optical Properties of CeNi_5_ Nanoscale Films

**DOI:** 10.1186/s11671-016-1477-7

**Published:** 2016-05-17

**Authors:** Radu Todoran, Daniela Todoran, Dania Racolta, Zsolt Szakács

**Affiliations:** Department of Economics and Physics, Technical University of Cluj Napoca, North University Center of Baia Mare, Dr. V. Babes Street, 62/A, Baia Mare, Romania

**Keywords:** CeNi_5_ thin films, Laser vaporization, Electrical conductivity, Electron band structure, 68.55.-a, 68.35.bd, 73.63.Bd

## Abstract

Rare earth compounds are interesting from both a theoretical point of view and for their applications. That is the reason why determining their optical and electrical properties deserves special attention. In this article, we present the conditions we obtained homogenous CeNi_5_ thin films of nanometer thicknesses. To achieve this goal, our method of choice was laser-induced vaporization, using short and modulated impulses, with electro-optical tuning for the quality factor. The layers that were deposited at a single laser burst had thicknesses between 1.5 and 2.5 nm, depending on the geometry of the experimental setup.

Structural and compositional studies of the nanoscale films were made using XRD. The temperature dependence of electrical conductivity was also determined. The following optical properties of the specimens were computed using the Kramers-Krönig framework and discussed: absolute reflection and transmission coefficients for a single wavelength and relative ones for the wide UV-VIS-IR spectra, spectral dependence of the refractive index, and extinction coefficient as real and imaginary parts of the complex refractive index. The valence band studies were made with X-ray photoelectron spectroscopy. All these determinations were well correlated and permitted the evaluation of the energy densities of states in the deeper bands, near the Fermi energy, and at the surface states.

## Background

The intermetallic compounds of rare earth metals with nickel are interesting from a theoretical point of view [1–4] as well as because of their applications in hydrogen storage as reversible metal hydrides. Cerium-based intermetallic compounds with Nickel have intermediate valence with strong hybridization between the 4f and conduction electrons [5–7] and properties of a Kondo lattice [8] and display enhanced Pauli paramagnetism [9] and spin-fluctuation behavior [10]. Cerium-based hydrogen storage alloys have been widely used because of their high energy density, high rate of discharge, and long charge-discharge cycle lifetime, while the effect of partial substitution of Ni with various metals (Cu, Fe, Co, Mn, Al, Si, Sn, Cr) has also been studied extensively [9–16].

## Methods

The energy spectrum of electrons in metal alloys strongly influences their optical properties. That is why optical response in the UV-VIS-IR region, which involves transitions between electronic bands from occupied states below the Fermi energy to empty states above, strongly contributes to the features in the experimental spectra. The surface states present in the thin films also influence the energy dispersion, drastically changing their properties. In this paper, we report the electrical and optical properties of the hexagonal CeNi_5_ thin films for different nanometer thicknesses until properties of the bulk material are recovered. Temperature-dependent studies of electrical conductivity are made. Electronic properties near the Fermi energy and deeper bands are correlated with X-ray photoelectron spectroscopy (XPS) studies.

The CeNi_5_ thin films were obtained using laser-induced vaporization from bulk powder compound. The bulk CeNi_5_ alloy was synthesized from 99.9 % pure initial metals in stoichiometric proportions by the classical method of electric arc melting in argon atmosphere at a low pressure of 10^−5^ Torr. The compounds were grinded in an agate mill. A quantity of 10–12 mg of powder with a granulation higher than 12 μm was used in the deposition cycles. The deposition substrate was glassed with dimensions suitable in the electrical determinations in the domain 80–300 K and optical measurements in a broad wavelength domain.

**Fig. 1:**
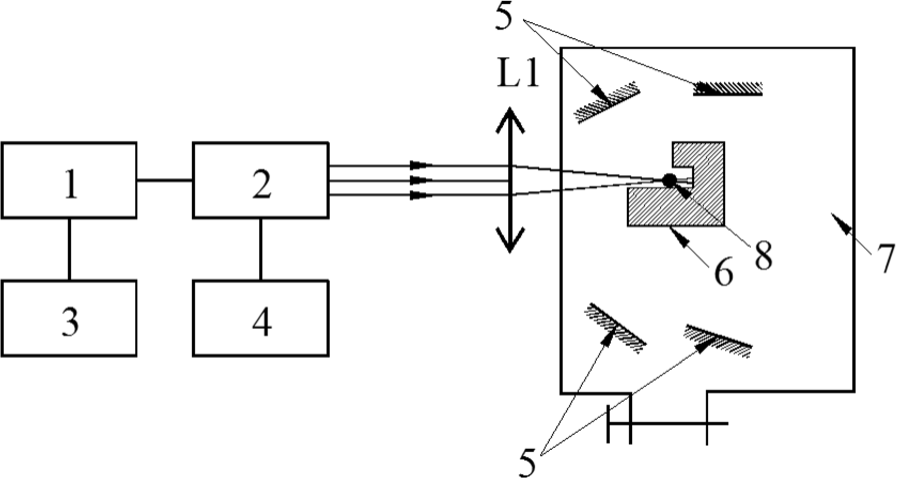
Schematic representation of the pulsed laser-induced vaporization system. *1* power supply unit of the laser, *2* the laser resonator containing the optically the active elements, *3* laser burst length control unit, *4* radiated power measurement unit, *L1* focusing lens with *F* = 220 mm, *5* deposition substrates, *6* support and cooling system of the bulk material, *7* vacuum chamber, *8* the bulk material to be vaporized

The schematic representation of our experimental film deposition setup can be seen in Fig. 1.

The laser had an Al_2_O_3_(Nd^3+^) ruby quantum generator (LGIN-503). Using quality factor modulation for the resonator, this laser source can function with an impulse length of 20–50 ns. Its laser burst frequency can be varied between 0.01 and 1 s. The energy density of the laser radiation can be varied using nonselective optical filters between 10^4^ and 10^9^ W/cm^2^. The pulse length that we used was 20 ns at a power of 3 × 10^7^ W with a frequency of around 10 Hz. The focusing of the laser beam was done using a spherical lens. The focus spot on the surface of the bulk powder had a diameter of 100 μm. The substrates were positioned at an angle of 10°–15° from the perpendicular direction of the vapor source at a distance of 3–5 cm. The angle and the distance influenced the thickness of the deposited thin films. A copper radiator was used to prevent the heating of the entire bulk CeNi_5_. The vaporization was made in a vacuum chamber at a pressure of 10^−5^ Torr (VUP-5). The wavelengths of the laser radiation were *λ*_1_ = 1060 nm and its second harmonic *λ*_2_ = 530 nm, which also lit the region of the sample that was exposed to its action. With these parameters, one could achieve instant vaporization while keeping the liquid phase for longer than 1.5 s.

Another vaporization method for the bulk powder taken into account was the one from inside a conically spun tungsten filament of 0.2-mm thickness while its temperature was kept at around 3600 °C. An optoelectronic system was used to maintain the temperature of the resistive heating source constant during the vaporization and between the cycles. This method was considered even before the laser vaporization. Although the success of the resistive vaporization was questionable from the start, it is a relatively cheap method. It also led us to some precious conclusions which were applied to the laser vaporization setup, a more expensive and complex one. The weakness of the vaporization method using a tungsten filament comes from the fact that the boiling temperatures of Ce and Ni are very different. This leads to the instability of the CeNi_5_ alloy at high temperatures, which tends to dissociate in its elemental components.

To determine the thicknesses of the nanoscale films, we used interferometric microscopy techniques (MII-5 microscope). The light source was changed to a sodium gas discharge lamp. The interpretation of interference figures permitted measurements of film thicknesses starting from 50 nm with a precision of 10 %. Lower thicknesses were determined by extrapolation. In the case of our CeNi_5_ films, the maximum theoretical thickness of 5 μm was not attained. Ellipsometric measurements were not applicable in our case because the optical functions *k*, *n*, *ε*_1_, *ε*_2_ must have been known a priori. Also, the amplitudes of the reflected electromagnetic waves *E*_*⊥*_ and *E*_*∥*_ differ less in the case of metallic thin films than for dielectrics or semiconductors.

The structure and phase composition of the deposited metallic nanoscale films was studied using XRD and the Cu K_α_ radiation (*λ* = 0.154 nm). In the phase identification process of our polycrystalline thin film samples, we searched for similarities between the obtained XRD peak positions and the peaks of the phases that are suspected to be present, but also compared them to the diffractogram of the bulk CeNi_5_ powder. The crystallite dimensions and micro-strains have also been determined this way.

Electrical conductivity measurements were made in the interval of 80–300 K using the four-probe method [17].

Spectral dependences of the reflectance *R*(*λ*) and transmittance *T*(*λ*) were measured in between 185 and 950 nm (Specord M40) and between 2.5 and 25 μm (Specord 75IR) using the spectrophotometers. The apparatus was adapted for measurements at an incidence angle smaller than 8°. The spectral resolution in the mentioned wavelength domains was better than 0.5 meV. Spectral calibration was made using amorphous quartz. This method was used because spectrometers do high-precision relative spectral measurements although they do not permit the determination for the absolute magnitude of the reflectance and transmittance that is necessary in the computations of the spectral functions.

**Fig. 2:**
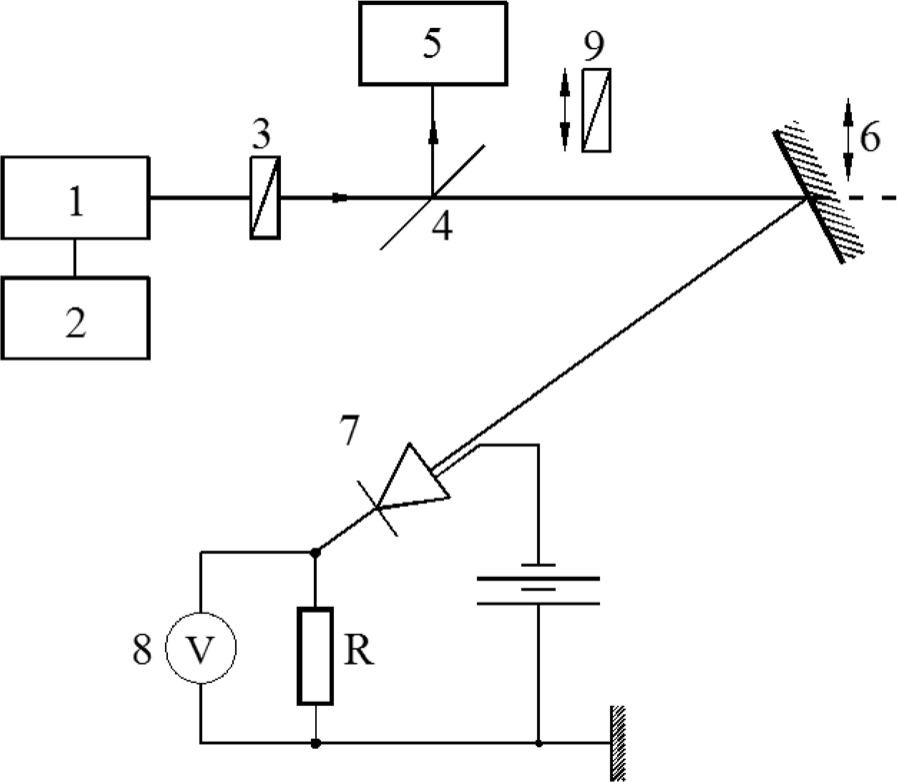
Schematic representation absolute reflectivity measurement setup. *1* He-Ne laser source, *2* laser source power supply unit, *3 λ*/4 crystalline quartz plaquette, *4* glass slide with a reflectivity of 0.04 at the selected laser wavelength, *5* laser radiation power meter, *6* the deposited film fixed on the goniometer, *7* Si photodiode, *8* digital voltmeter, *9* Glan-Thompson polarizer

To compute the absolute reflectance and transmittance, it is enough to determine it at a single wavelength in the spectral domain under consideration and then use relative determinations. This was done using an He-Ne laser (LGN-215-type) with *λ* = 632.8 nm, *P* = 40 mW at an incidence smaller than 3°, with the schematic representation of the experimental setup depicted in Fig. 2.

The time stability of the laser source was determined using a laser power radiation meter (ILI-5). The prepared samples were installed on a goniometer (GS-5) with a precision for the incidence angle of 1.5ʺ. The plane-polarized light emitted by the laser was transformed in circularly polarized radiation using a *λ*/4 crystalline quartz plaquette. The incident and reflected radiation was measured with a Si photodiode (FD-10K) and a digital voltmeter (V7-21). The components of the reflectance *R*_*⊥*_ and *R*_*∥*_ were separated using a polarizing filter. The reflectance was determined as the quotient of the reflected laser intensity from the deposited film surface and the direct incident radiation intensity, both being measured using the same laser power radiation meter. This device was calibrated prior to its use with an Ag mirror (*R* = 0.94 ± 0.0005).

The spectral dependences of the refractive index *n(ωħ)* and extinction coefficient *k(ωħ)* for the deposited thin films were obtained from the reflection spectra in the Kramers-Krönig framework [4, 18–21]. Knowing that this formalism works best when the spectral data covers a wide domain, we proceeded to extrapolate *R(λ)* in the UV region down to *λ* = 125 nm using an 1*/a*^*υ*^-type law. Here, *a* is a dimensionless coefficient which was determined using two lines in the Lyman series of the hydrogen lamp with a LiF window and *υ* is the photon frequency.

The XPS spectra were recorded using an XPS spectrometer (PHI 5600) and its collimated monochromatic Al K_α_ radiation (1486.6 eV) with the FWHM of this line of 0.3 eV [22, 23]. In this way, only the electrons in a layer of approximately 2 nm on the surface of the sample give useful information in the spectral determinations. The samples were cut in high vacuum to avoid surface contamination. Ablation was made using Ar^+^ ions, accelerated at very low potentials, so that the first layer of the film is cleaned but the surface states remain unmodified. Then, the sample was introduced in the main experimental chamber, where the pressure was as low as 10^−19^ Torr.

## Results and Discussion

### Thickness, Composition, and Phases

**Fig. 3:**
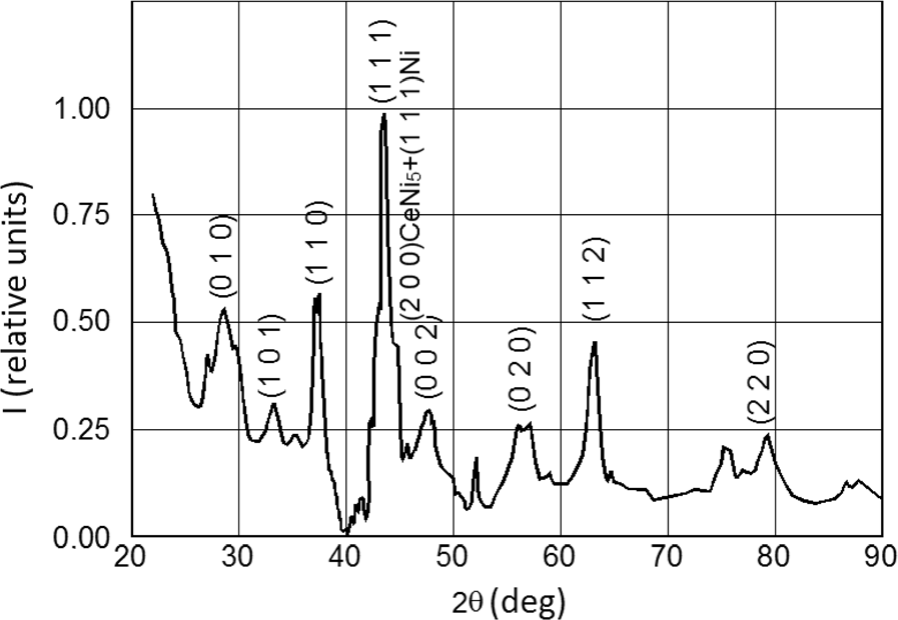
XRD pattern of the 200-nm thick film deposited using resistive heating. One can observe a much higher intensity of the peak marked (200)CeNi_5_ + (111)Ni than in the case of the pure CeNi_5_ patterns

The first films studied by us where the ones deposited by resistive heating vaporization. As expected, and can be seen in Fig. 3, in their XRD patterns, a peak increase appeared at 2*θ* = 44.6°, reflected from the crystalline planes (111) of Ni and superimposed over the (200) peaks of CeNi_5_, proving the dissociation of the initial CeNi_5_ bulk powder, increasing the peak intensity. This vaporization method also led to very low deposition efficiencies of around 3 %. But from this study, we concluded that the whole bulk mass would be vaporized in more than 2 s, later determining the number of impulses needed to complete the laser-induced vaporization process. No further investigations of these films were conducted.

**Fig. 4:**
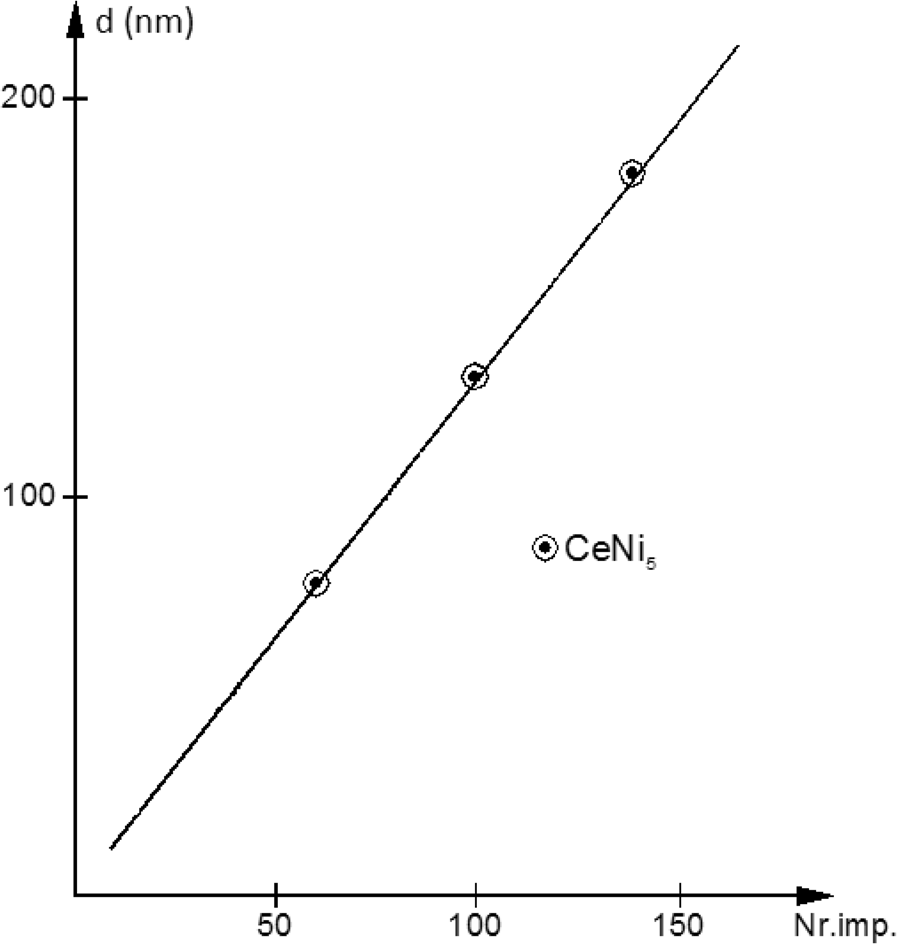
Dependence of the CeNi_5_ film thickness vs. the number of laser impulses

In the case of laser-induced vaporization, studying the thickness of the deposited films vs. the number of laser impulses, presented in Fig. 4, a good linear dependence can be noticed. The average thickness per laser impulse was between 1.5 and 2.5 nm depending on the angle and distance between the bulk and the deposition substrate. This good linearity allowed us to extrapolate and find the approximate thickness of the films with *d* < 50 nm, below the possibilities of the microscope technique that was used.

**Fig. 5:**
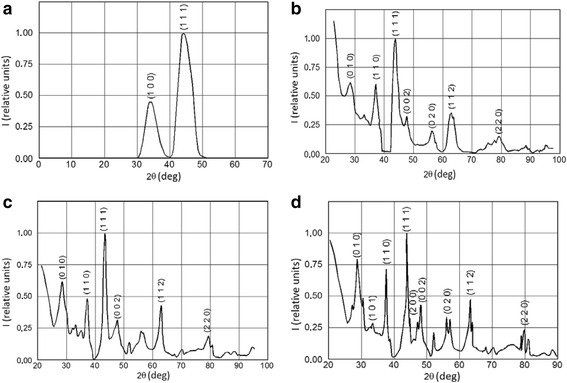
XRD patterns of the deposited films with thickness of **a** 6 nm, **b** 45 nm, **c** 220 nm, and **d** of the primary bulk powder as a reference

Using this vaporization technique, the XRD patterns, displayed in Fig. 5, do not exhibit the peak increase associated with crystalline planes of Ni anymore. For the film of *d* = 6 nm, only the reflections on the (100) and (111) family can be differentiated from the substrate signal and are quite largely broadened. The signal from the substrate can also be identified in all cases due to the low attenuation of the films. The CaCu_5_ structure of the films can rapidly be verified using as a comparison basis the diffraction pattern obtained from the bulk that can be observed in Fig. 5d.

At low film thicknesses, one can observe wider peaks compared to the images obtained from the bulk. It is well known that this is an effect due to an instrumental factor, to the small dimensions of the analyzed crystallites and the micro-strain that appears in these cases [24]. The instrumental factor was determined using the FWHM of crystalline Si powder at the 2*θ* = 33.17° peak where the doublet radiation effects were also taken into account. Because the obtained peaks can be well approximated with a Gaussian distribution, the corrected FWHM obeys the formula:

1 $$ {\alpha}^2={\left(\frac{0.9\lambda }{D \cos \kern.1em \theta}\right)}^2+{\left(4\frac{\varDelta d}{d}tg\theta \right)}^2, $$

where *λ* = 0.154 nm is the wavelength of the used X-ray radiation, *θ* is the diffraction angle, *d* is the distance between the crystallographic planes (111) in the bulk which we took into account, *D* is the average dimension of the crystallites, and the micro-strain has the form:

2 $$ \frac{\varDelta d}{d}=\frac{d-{d}_0}{d}, $$

with *d*_0_ being the distance between the same considered planes in the deposited crystallites. We obtained an average dimension of the crystallites of *D* = 5 nm for the 45-nm thick film. The micro-strain did not exceed 5 % in this case.

### Electrical Conductivity

The temperature dependence of the electrical conductivity of thin metallic films generally obeys the following formula [25]:

3 $$ \sigma ={\sigma}_1+{\sigma}_2(T), $$

where the first term is the conductivity conditioned by the scattering on static defects in the film and the second term depends on the mechanism of electron-phonon interaction.

**Fig. 6:**
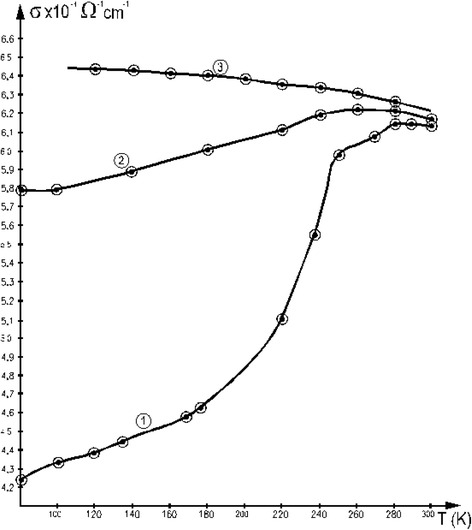
Temperature dependence of electrical conductivity of thin films with *1 d* = 45 nm, *2 d* = 110 nm, and *3 d* = 220 nm

The temperature dependence of the conductivity is represented in Fig. 6 for thicknesses of 45, 110, and 220 nm, respectively.

The thermal coefficient of the conductivity is negative for thicknesses bigger than 220 nm, confirming the metallic behavior of this thin layer. For thicknesses lower than approximately 110 nm, the behavior changes. Below 250 K, this coefficient is positive and increases inversely with the film thickness. This shows the well-determined activating character of the temperature. Above the value of 250 K, an exponentially increasing interaction of the charge carriers with the phonons is displayed with the temperature increase. At these temperatures, the thin films behave like the bulk and have a negative temperature coefficient.

For an explanation of the behavior presented above, in the case of the thinnest films, one should resort to the idea of insular deposition of the layers. The number of surface states in the case of discontinuous films is very high compared to the bulk. The charge carriers are captured on these states, and the level occupancy number of the valence band decreases. Because of this, the number of possible transitions near the valence band increases, opening the path to explore this energy region by optical means. This observation is especially important near the Fermi energy since most of the physical properties depend on the density of the states near it. The thermal energy of the charge carriers increases with the temperature, so inter-insular hopping is possible. Inter-insular tunneling is also an important factor which must be taken into account. Our thin-film deposition strategy considered the abovementioned deductions, and we deliberately wanted to obtain discontinuous thin films in our processes.

In the case of insular film depositions, one must take into account another energy gap, the one between the isles denoted by *ΔE*_*a*_, so that the conductivity in formula (3) must be completed with a third, temperature dependent, exponential term:

4 $$ {\sigma}_3={\sigma}_0{e}^{-\frac{\varDelta {E}_a}{2{k}_BT}}, $$

where *σ*_0_ is the electrical conductivity extrapolated to 0 K.

**Fig. 7:**
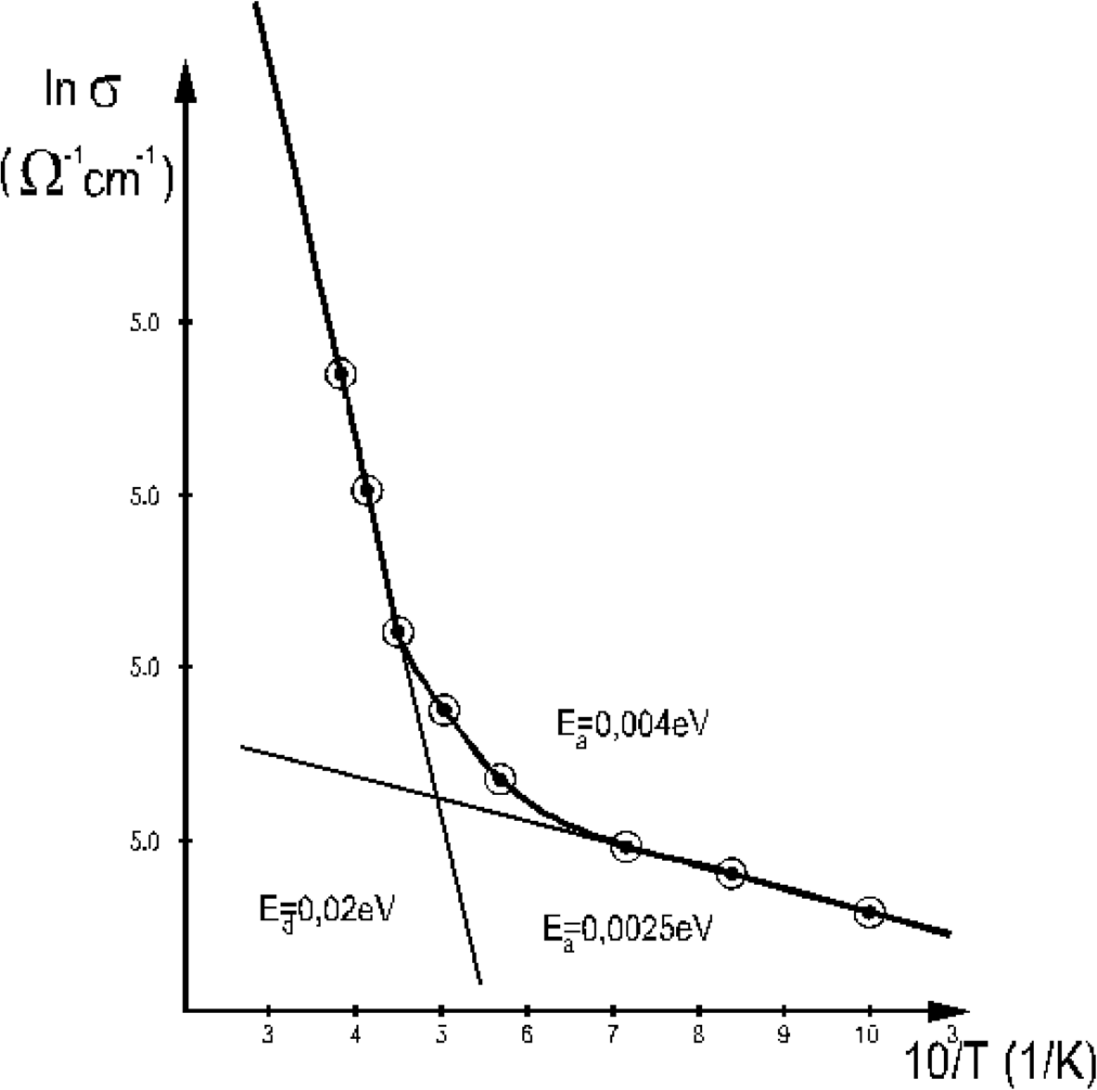
The logarithm of the conductivity vs. the inverse of the temperature in the case of the film with *d* = 60 nm

Because of this, a representation of the conductivity in a logarithmic scale vs. the inverse of the temperature gives more insight of the processes involved. This graph can be viewed in Fig. 7.

One can deduce that, for temperature domain 100–140 K, the activation energy is approximately 4 meV, while for temperatures between 200 and 300 K, it increases to 20 meV.

In conclusion, the deposited films display three different characters. The thinnest films are formed by small isles, which are short-circuited by bigger ones as the film thickness increases, until, in the last phase, all isles are short-circuited and the properties of the bulk are recovered in the “thickest” films. The insular part of the deposited films does not play a major role in the properties of the thickest films. That is why the most interesting properties to be studied are in the domain of the thinnest films.

### Reflectance and Transmittance

**Fig. 8:**
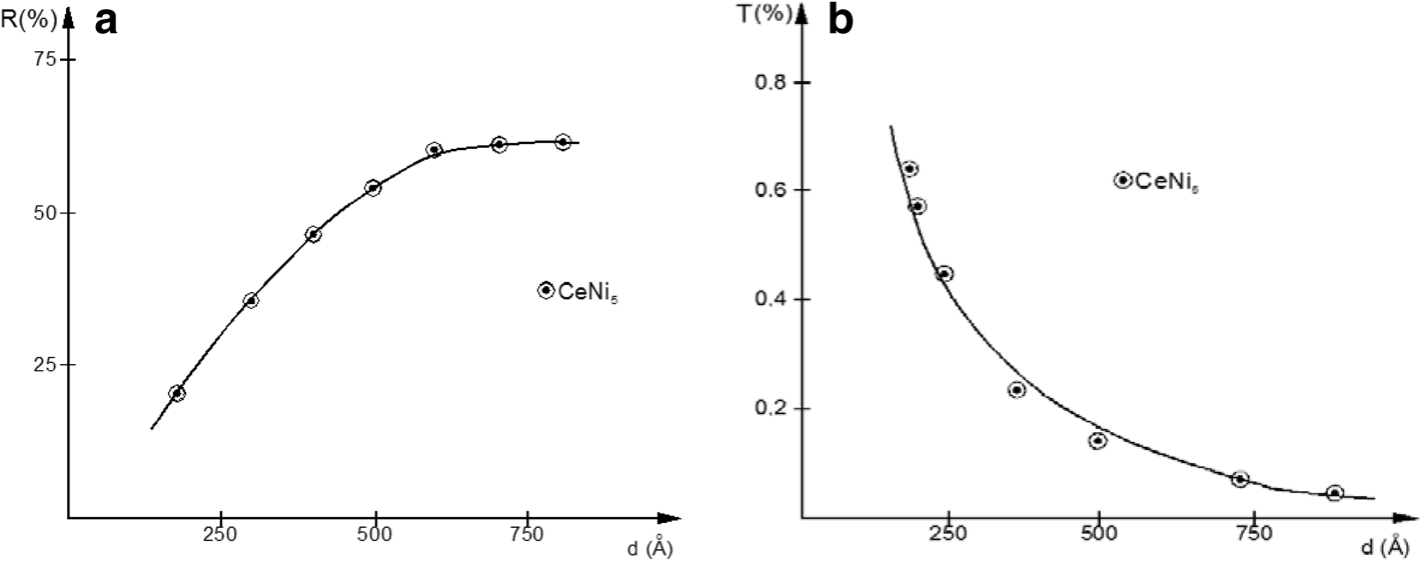
Layer thickness dependence of the **a** absolute reflectance and **b** absolute transmittance for the deposited thin films at the *λ* = 632.8 nm radiation of the used He-Ne laser

Figure 8a, b presents the thickness dependence of the absolute reflectance and, respectively, absolute transmittance of the films for the selected He-Ne laser wavelength of *λ =* 632.8 nm. The reflectance displays a slower-than-linear increase up to the thickness of *d* = 60 nm, after which it can be considered to stay constant. Its plateau is at a value of around 62 %, equal with the one for the bulk.

Studying the film-thickness dependence of the transmittance, one can observe a sufficiently good fit of the equation [24]:

5 $$ T=\frac{{\left(1-R\right)}^2{e}^{-\alpha d}}{1-{R}^2{e}^{-2\alpha d}} $$

where *α* is the optical absorption coefficient for the specified wavelength.

**Fig. 9:**
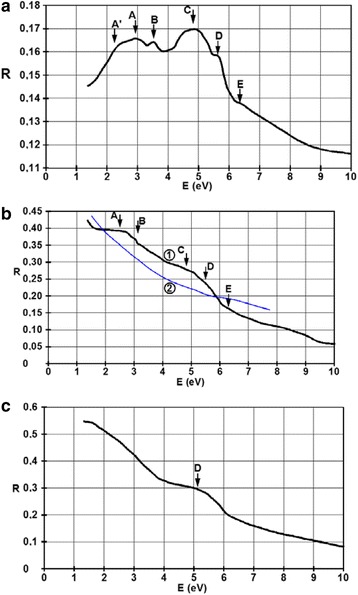
Spectral dependence of the reflectance for the films with thicknesses of **a** 6 nm, **b** 27 nm, and **c** 45 nm. In part **b**, the experimental data, plotted with *black line*, is denoted by *1* and the results computed from the classical Drude model, plotted with a *blue line*, are denoted with *2*

Figure 9 shows the spectral dependence of the reflectance for the films with thicknesses of 6, 27, and 45 nm in the 1–10 eV domain.

The band structure of the *d* = 6 nm film has its local maxima at (A) 2.7 eV, (B) 3.5 eV, (C) 4.8 eV, (D) 5.5 eV, and (E) 6.3 eV. In the domain below 2.1 eV, its increasing tendency is a characteristic of dielectrics and semiconductors. Here, the photon energy tends to equal the band gap. One can say that the charge carriers are captured on the surface states. At low film thicknesses, this kind of spectral dependence is given by the electronic transitions from totally occupied electronic bands to partially occupied ones or to surface states above the Fermi level.

When the thickness of the film increases to *d* = 27 nm, one can see that the maximum denoted by E disappears almost completely. Maxima C and D form a single band with its dominant value at approximately 5 eV. The A and B maxima are barely distinguishable and form a band in the 3 eV region. Besides the optical transitions from the deepest bands, there is also absorption due to the free charge carriers. The blue line in Fig. 9b represents the electron-photon interaction computed from theory using the classical Drude model. The influence of the free electrons can be seen also from the overall increase in the reflectance values over the 1.5–6.5 eV domain.

For the film with a thickness of *d* = 45 nm, only the D characteristic survives, while the rest of the plot resembles more to the properties of the bulk.

### The Refractive Index *n* and the Extinction Coefficient *k*

**Fig. 10:**
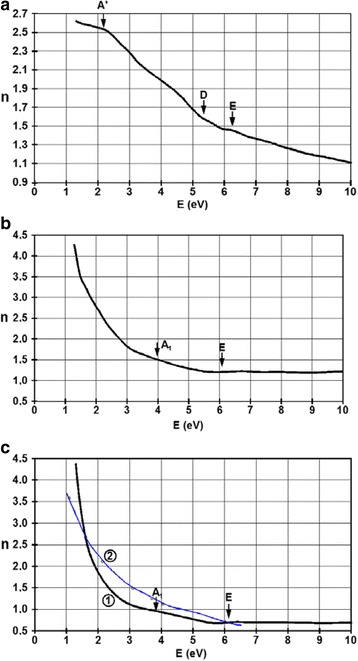
Spectral dependence of the refractive index for the films with thicknesses of **a** 6 nm, **b** 27 nm, and **c** 45 nm. In part **c**, the experimental data, plotted with *black line*, is denoted by *1* and the results computed from the classical Drude model, plotted with a *blue line*, are denoted with *2*

Figure 10 presents the spectral dependence of the refractive index for films with thicknesses of 6, 27, and 45 nm. These optical coefficients were computed using the Kramers-Krönig relations.

The values of the refractive index decrease with the photon energy. The maxima denoted A, D, and E in the spectral dependence of the reflectance are also present. The low values of *n* underline again that photons interact with tightly bound electrons. In the case of the film with 27 and 45 nm, the abrupt increase below 3 eV indicates again a much higher concentration of the electrons than the holes in this band and the interaction of photons with these electrons. One can observe two weak and wide maxima denoted A_1_*ωħ* ≈ 4 eV and E shifted a little bit towards *ωħ* ≈ 6.3 eV. For high energies, above 5 eV, the refractive index almost does not display any variation with the photon energy. In the case of the thicker films, all values are bigger than the 6-nm thick film.

Computing the theoretical curve, one used the proportionality of the refractive index with the square root of the wavelength. This formalism be applied with success in the case of tight-binding electrons and a parabolic conduction band, for example, Ag, Au, or Al. One can observe a much higher increase rate of our experimental curves than as the tight-binding theory predicts.

**Fig. 11:**
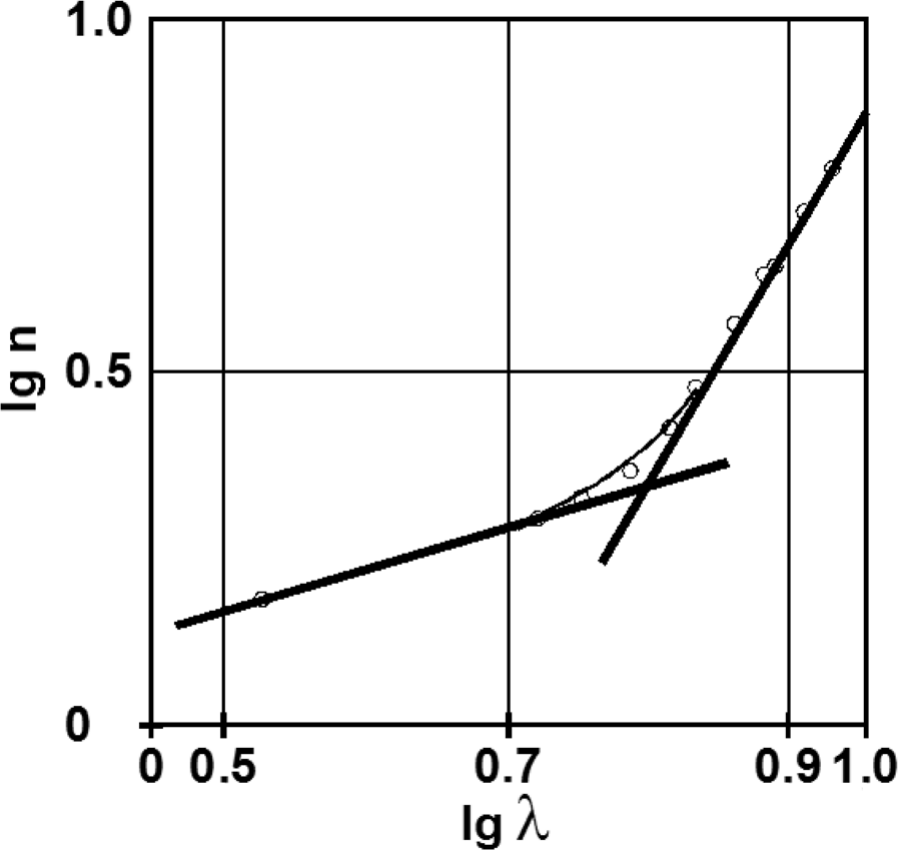
Rescaled lg-lg plot of the wavelength dependence of refractive index in the long-wavelength VIS-IR region

Following these remarks and representing a lg-lg plot of the *n(λ)* dependence for a film with thickness of 45 nm, an interesting feature can be observed in the long-wavelength VIS-IR domain, presented in Fig. 11. There are two different slopes for the interpolating lines, which can be explained by the non-parabolic shape of the energy bands. The very rapid increase in the IR region has a slope much higher than 1, confirming the photon-free electron interaction.

**Fig. 12:**
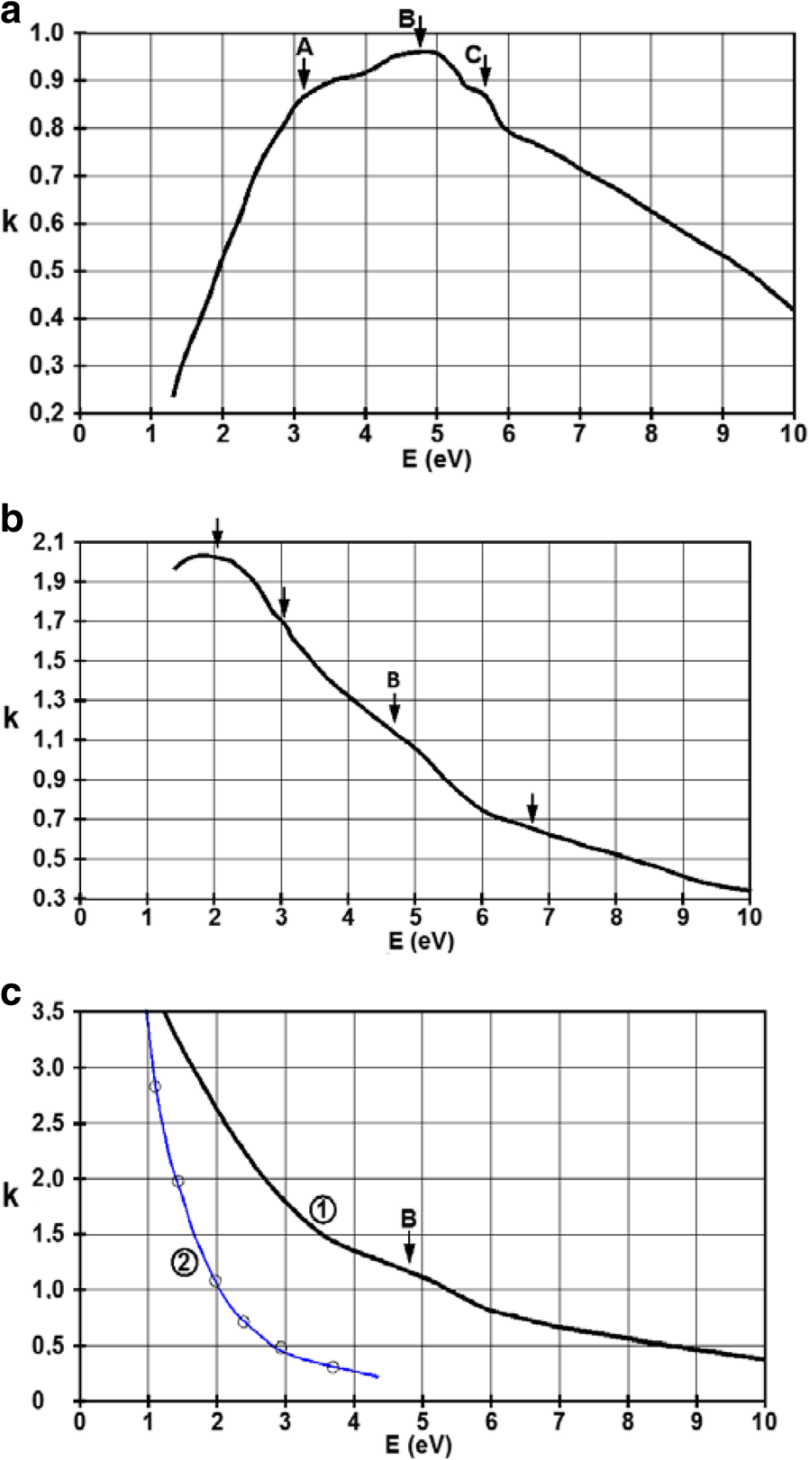
Spectral dependence of the extinction coefficient for the films with thicknesses of **a** 6 nm, **b** 27 nm, and **c** 45 nm. In part **c,** the experimental data, plotted with *black line*, is denoted by *1* and the results computed from the classical Drude model, plotted with a *blue line*, are denoted with *2*

Figure 12 presents the spectral dependence of the extinction coefficient for films with thicknesses of 6, 27, and 45 nm, computed, as before, using the Kramers-Krönig formalism.

**Fig. 13:**
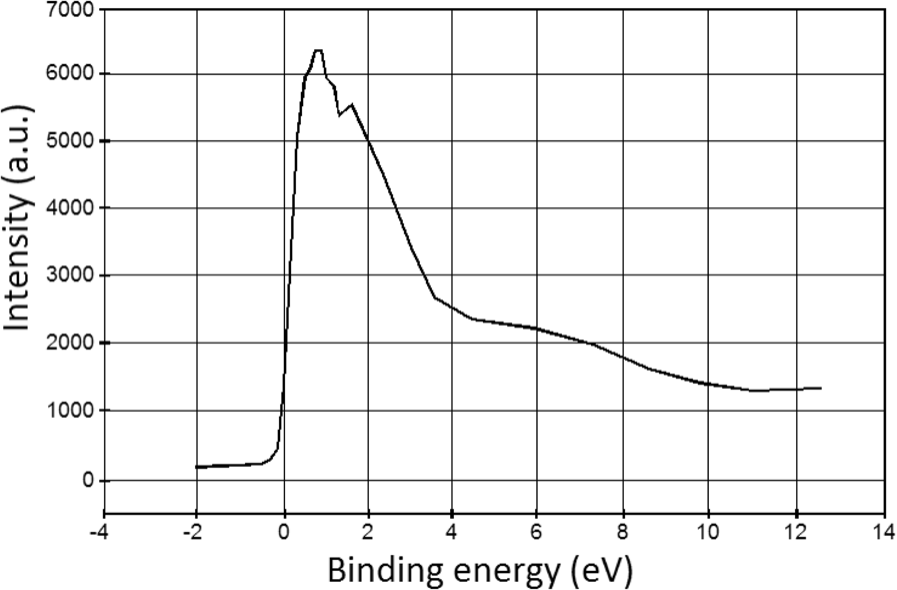
XPS spectrum of the valence band for the 6-nm thick film

In the case of *d* = 6 nm, we obtained the characteristic shape for materials with thermally excited conductivity. This also indicates that the film has an insular structure. The region below the energy of 4 eV can be interpreted using photon absorption between two bands separated by a gap. The width of this gap can be obtained extrapolating the function to *k* = 0, so its approximate value is 1 eV. The decreasing behavior of the extinction at *ωħ* > 6 eV coefficient may be determined by a lower density of states in the filled electronic band. In the domain 3–6 eV, the maxima are located at (A) 3.4 eV, (B) 4.6 eV, and (C) 5.5 eV, indicating a high DOS in this region. A good correlation can be seen between the XPS spectra of the valence band and the shape of *k(ωħ)* in Fig. 13.

Important changes take place in the behavior of the extinction coefficient in the case of a film with a thickness of 27 and 45 nm. Extinction values are higher and rapidly increasing for the wavelengths above 250 nm. This can be a result of higher DOS values near the Fermi energy. It must also be mentioned that, beside the photon-free electron interaction, there are transitions which can be easily noticed, around the energy value of B *ωħ* ≈ 5 eV. These are signs of transitions from much deeper-laying bands. This wide forbidden band, dominant for *d* = 6 nm, transforms into a gap as the thickness of the film increases. In Fig. 12c, the theoretical curve (2) was computed using the interaction of the photons with free charge carrier based on the Drude model. It can be observed that this interaction influences the values of *k(ωħ)* in the NIR domain, while for the UV-VIS interval, the description must be done resorting to the tight-binding description, as in the case of the presented CeNi_5_ films. A better theoretical description of metallic films with thicknesses of a few times the lattice constant that might be considered would be perhaps the one of electrons moving in two dimensional lattice structures.

For energies higher than 6 eV, the optical functions do not depend on the film thickness, so the interactions of the photons are with tight-binding electrons.

## Conclusions

In the present article, we report successful preparation of CeNi_5_ nanoscale thin films obtained from bulk CeNi_5_ powder using laser-induced vaporization, with short and modulated impulses and electro-optical tuning for the quality factor. The film thickness follows a liner increase with the number of laser bursts. Each layer has a thickness of 1.5–2.5 nm, depending on the position of the deposition substrate in the experimental chamber. Structural and compositional studies of our nanoscale layers were made using XRD and exhibiting a single-phase diffractogram, without the separation of Ni crystallites. The micro-strain and the average crystallite dimensions were determined; the temperature dependence of electrical conductivity was also discussed. We obtained three different deposition characteristics. The thinnest films, below 15 nm thickness, display small insular characteristics, temperature-activated conduction properties with inter-insular hopping, and a high carrier density in the surface states. In the intermediate thickness region of 27–110 nm, less surface states are present because some small islands are short-circuited, so that the inter-insular hopping energy decreases. The thicker films, with *d* > 150 nm, display a bulk-like metallic behavior, almost all islands are short-circuited, and the influence of the first few layers is negligible. The reflection and transmission dependence on the film thickness also supports our previous observations. From the spectral distributions of reflectivity, we deduced the energy band distribution in the case of different thicknesses, receiving information about the inter-band hopping energies and band-filling factors. Comparison with the theoretical classical Drude model, electron-photon interaction was also discussed, and the tight-binding model was also used to explain the spectral behavior. The lg-lg representation of *n(λ)* displays a non-parabolic shape of the bands in the long-wavelength region. The spectral dependence of the extinction coefficient was compared to the XPS studies, giving a good correlation of the conclusions. This underlines the insular deposition of our films and enhances the understanding of the energy and forbidden band structures. Our electrical, optical, and XPS determinations show good correlations and permit the evaluation of the hopping energies, energy densities of states in the deeper bands, near the Fermi energy, and at the surface states besides deposition geometries of the layers.
